# Challenges and opportunities for characterizing cognitive aging across species

**DOI:** 10.3389/fnagi.2012.00006

**Published:** 2012-09-12

**Authors:** Erik D. Roberson, R. Anthony DeFazio, Carol A. Barnes, Gene E. Alexander, Jennifer L. Bizon, Dawn Bowers, Thomas C. Foster, Elizabeth L. Glisky, Bonnie E. Levin, Lee Ryan, Clinton B. Wright, David S. Geldmacher

**Affiliations:** ^1^Departments of Neurology and Neurobiology, Evelyn F. McKnight Brain Institute, University of Alabama at BirminghamBirmingham, AL, USA; ^2^Department of Neurology, Miller School of Medicine, Evelyn F. McKnight Brain Institute, University of MiamiMiami, FL, USA; ^3^Department of Psychology, University of ArizonaTucson, AZ, USA; ^4^Department of Neurology, University of ArizonaTucson, AZ, USA; ^5^Evelyn F. McKnight Brain Institute, University of ArizonaTucson, AZ, USA; ^6^Department of Neuroscience, University of FloridaGainesville, FL, USA; ^7^McKnight Brain Institute, University of FloridaGainesville, FL, USA; ^8^Department of Clinical and Health Psychology, University of FloridaGainesville, FL, USA; ^9^Department of Psychology, University of MiamiMiami, FL, USA; ^10^Department of Epidemiology and Public Health, University of MiamiMiami, FL, USA; ^11^Neuroscience Program, University of MiamiMiami, FL, USA

**Keywords:** cognition, aging, memory, human, animal models

## Abstract

The gradual decline of cognitive ability with age, even in the absence of overt brain disease, is a growing problem. Although cognitive aging is a common and feared accompaniment of the aging process, its underlying mechanisms are not well understood and there are no highly effective means to prevent it. Additional research on cognitive aging is sorely needed, and methods that enable ready translation between human subjects and animal models stand to provide the most benefit. Here and in the six companion pieces in this special issue, we discuss a variety of challenges and opportunities for studying cognitive aging across species. We identify tests of associative memory, recognition memory, spatial and contextual memory, and working memory and executive function as cognitive domains that are age-sensitive and amenable to testing with parallel means in both humans and animal models. We summarize some of the important challenges in using animal models to test cognition. We describe unique opportunities to study cognitive aging in human subjects, such as those provided by recent large-scale initiatives to characterize cognition in large groups of subjects across the lifespan. Finally, we highlight some of the challenges of studying cognitive aging in human subjects.

## Introduction

One of the great successes of the last century was a dramatic increase in life expectancy, which increased in the United States from 49.2 years in 1902 to around 78 years today (Congressional Research Service, 2006). With the many benefits of increased longevity, however, come the burdens of aging, among the most feared of which is the potential decline of cognitive ability. The effects of aging on cognition have traditionally been divided into three trajectories. The most severe decline is associated with age-related diseases, such as Alzheimer's disease and other neurodegenerative dementias. At the other end of the spectrum are those who maintain high levels of cognitive function as the age, often termed successful aging (Rowe and Kahn, 1987). But the most common trajectory is somewhere between these two extremes: those who escape from succumbing to age-related disease but still experience a gradual impairment of cognition that is sometimes called “normal aging” and will be referred to here as cognitive aging. Cognitive aging begins as early as the fifth decade (Singh-Manoux et al., 2012) and can negatively impact quality of life. The rapidly growing population of older Americans makes it critical that we develop new strategies to treat and prevent cognitive aging.

Cognitive aging can be studied in both human subjects and animal models, and each approach presents unique advantages and disadvantages. Without doubt, the gold standard for cognitive aging studies is research with human populations. Human subject research is necessary to describe the epidemiology of this problem, define which cognitive domains are most susceptible to decline with aging, understand the cognitive processes mediating these declines, and to test potential interventions. But human studies have important constraints, particularly the limited ability to test hypotheses regarding neural substrates of age-related cognitive decline through experimental manipulations. Animal models are much better suited for these experiments, since they allow for neuropathological examination at different time points and for experimental manipulations that aren't possible in human subjects. The issue, of course, is identifying animal models and outcome measures that are most relevant to human cognitive aging and have the best predictive value for identifying therapeutic strategies. Thus, there is a need for complementary approaches that allow facile translation of findings across species.

To address these issues, the McKnight Brain Research Foundation (tmbrf.org) convened a working group of scientists from the four McKnight Brain Institutes, which are devoted to studying cognitive aging. This group was charged with evaluating the current challenges and opportunities for studying cognitive aging across species. In the six companion pieces in this special issue, we make recommendations on suitable assays for characterizing cognitive aging in human populations and both rodent and non-human primate models. Here, we summarize our findings and review overarching issues regarding studies of cognitive aging across species.

## Opportunities with animal models

In reviewing the literature on animal models of cognitive aging, we set three criteria by which to select models and tests for consideration. First, the test must probe a cognitive domain that is known to decline with aging in both humans and in the animal model. Second, there must be some understanding of the anatomic substrate of the test, either a circumscribed region or a distributed network, with the rationale that a cognitive test in animal models is more likely to have relevance to human subjects if it is mediated by a conserved anatomical region/network. For this reason, despite the fact that some lower organisms have age-dependent declines on learning and memory tasks, our discussion is limited for the most part to mammalian models, primarily rodents and non-human primates, which have brain networks homologous to humans. Third, the ideal outcome measures should be those that can be evaluated in both human subjects and animal models using tests that bear as much similarity as possible, again to maximize cross-species predictive validity.

Applying these criteria, we identified four cognitive domains that pose excellent opportunities for studying cognitive aging in animal models. First, associative memory can be assessed by classical eyeblink conditioning, is age-dependent, and relies on both cerebellar and hippocampal circuits across species (Engle and Barnes, 2012). Second, recognition memory can be assessed by object recognition tasks, is age-dependent, and relies on the perirhinal cortex across species (Burke et al., 2012, which includes further discussion of cases in which the hippocampus proper may also be involved, e.g., Reed and Squire, 1997; Zola and Squire, 2001). Third, spatial and contextual memory can be assessed with multiple tasks, is age-dependent, and relies on the hippocampal formation across species (Foster et al., 2012). And fourth, working memory and executive function can be assessed with a variety of cross-species tasks, are age-dependent, and rely on prefrontal cortex across species (Bizon et al., 2012). Details on the history of these cognitive domains, the evidence for their conserved anatomy and age-dependence across species, and strategies for testing them in animal models are presented in the companion articles in this issue.

## Challenges with animal models

While the availability of tests for associative, recognition, spatial/contextual, and working memory in animal models poses tremendous opportunities, there are a variety of challenges to these studies. In particular, there are many external variables that affect performance on these tests and must be carefully controlled to generate reliable and reproducible data.

### Effects of diurnal variation and timing

The light cycle timing of vivarium facilities is a major factor in several lines of animal research, including neuroscience and endocrinology. By default, many investigators who study nocturnal rodents use a standard light dark cycle (LD) in synchrony with work schedules (12 h light starting at 6 am and 12 h dark starting at 6 pm, termed a 12:12 LD cycle). Another popular alternative is a 14:10 LD cycle. Because rodents are in the inactive phase of their circadian rhythm during the daylight hours, some behavioral assays are better administered during the dark phase of the cycle (Hossain et al., 2004). Inverted light cycles, i.e., 12:12 DL, allow performing nocturnal experiments during normal working hours. However, careful attention must be paid to maintaining darkness during dark cycle experiments, as even minimal amounts of light during the dark cycle can disrupt the circadian rhythms with downstream effects on many neurophysiological parameters (Reiter, 1991). This variable is not an issue, of course, with non-human primates, who have circadian patterns much like those in humans.

### Effects of pre-handling and test order

On some tests, naïve rodents behave differently than rodents that have been handled or have previously undergone other behavioral assays (Holmes and Rodgers, 1998; Võikar et al., 2004). Thus, protocols for pre-handling before testing and standardization of test order become important. Generally, rodents should be handled daily for several days prior to experimentation. The goal of animal pre-handling is to acclimatize the animals to being picked up, restrained, and placed in position. Further, daily handling is particularly important in the context of aging with regard to minimizing stress and to provide opportunities to identify pathophysiological changes (e.g., tumor growth, urinal plugs, etc.). When multiple investigators are involved in animal studies, consistent handling parameters should be observed and all investigators should handle the animals regularly before experimentation. Adaptation of non-human primates to research personnel, primate holding chairs in which behavioral tests are administered, and the experimental routine of the study is also crucial for reducing variability among animals in any age group.

### Effects of single vs. group housing

Rodents are social animals and the degree of social interactions can have a profound effect on cognitive parameters. For example, animals socially isolated after weaning can perform better in the water maze (Wongwitdecha and Marsden, 1996). Therefore, the degree and timing of social isolation (single housing) should be clearly indicated. This holds true for non-human primates as well. One of us (Carol A. Barnes) has successfully pair-housed opposite-sex pairs for behavioral and electrophysiological experiments, and some labs pair-house same-sex, mature female monkeys or even groups of up to eight young males (Barnes and Baxter, personal communication).

### Effects of source/vendor and housing conditions

Rodent contextual fear conditioning results can vary between mice of the same strain purchased from different vendors (Curzon et al., 2009). In aging research, most groups rely on the NIA rodent aging colony, thus minimizing vendor differences. However, some groups maintain their own colonies, particularly for the study of transgenic lines. The details of colony maintenance can affect animal behavior, as each vivarium facility has unique characteristics that can contribute to inter-institutional variability. Difficult to control issues such as noise levels and building vibrations can contribute to chronic stress and affect both the aging process and learning and memory. Further, the effect of the local climate (e.g., heat and humidity in Florida) can influence animals during transport and housing in older facilities. Controllable factors include frequency of cage changes, animal and cage density, chow, light cycle, and the use of ventilated cages.

A number of National Primate Research Centers (e.g., California, Oregon, Yerkes, etc.) breed and maintain rhesus macaques and have detailed records of health and exposure to traumatic incidents across their lifespan.

### Effects of background strain

Rodent experiments can be performed on different inbred and outbred background strains, which can vary considerably in their abilities on cognitive tasks (Crawley et al., 1997). This emphasizes the importance of a consistent genetic background, and if one is studying age-related decline in ability, a background strain that is capable of performing the task at young ages. For non-human primates, while a direct experimental comparison of cognition has not been conducted on Indian- versus Chinese-derived rhesus macaques, there appear to be notable differences in temperament between these genetic backgrounds, which can, of course, affect cognitive outcomes.

As discussed below and in the companion article by Geldmacher et al. (2012) an important consideration in screening human subjects for cognitive studies is the identification of any sensory abnormalities (e.g., hearing or visual impairment) that might impair task performance independent of cognitive ability. Similar issues arise in animal model studies of cognitive aging, as some background strains develop age-dependent sensory abnormalities. For example, C57BL/6 mice display age-related hearing loss (Johnson et al., 1997) that can affect tests based on auditory cues. For non-human primates, useful tests of visual and auditory function across aging animals can be conducted using standard visual-evoked potential tests, tests of auditory brain responses, as well as more standard exams.

### Effects of specific protocol parameters

In addition to these general considerations, most tasks have specific aspects of the protocol that can affect results significantly, such as the number and spacing of trials. Key parameters for each task are discussed in the companion articles.

### Challenges with interpretation of behavior data

In some cases, it can be challenging to relate certain findings in animal models to human disease. For example, increased time spent exploring the open arms of the elevated plus maze could reflect decreased anxiety, increased risk taking, disinhibition, or hyperactivity. Obviously, these interpretive challenges are greater with tasks that have less relation to those used with human subjects. To mitigate this issue, we have tried to focus on tasks with as much “face validity”, or similarity in task performance between human subjects and animal models, as possible.

## Opportunities with human subjects

While experimental approaches have provided a wealth of information on age-related cognitive changes, the field would clearly benefit from the expansion of standardized research approaches to cognitive assessment in human aging studies. Several collaborative efforts have been developed to pursue these over the last decade, and each has strengths and weaknesses when viewed from the perspectives of (1) characterizing longitudinal changes within aging populations, (2) characterizing individual differences within aging populations, (3) aligning with the phenomenology and design of experiments to study cognitive aging in non-human species, and (4) adaptability for use by physicians and other health care providers who must differentiate the effects of healthy cognitive aging from incipient neurologic disease.

One these collaborations is the “NIH Toolbox for Assessment of Neurological and Behavioral Function” (www.nihtoolbox.org) which was established by the NIH to develop a common set of instruments to measure cognitive, emotional, motor and sensory function across diverse cultural, ethnic and geographic groups (Gershon et al., 2010). Participants range from age 3 to 85. From the cognitive aging perspective, a major shortcoming of the initial studies developing the toolbox is the allocation of adults from age 18 to 69 in a single age group, with another group ranging in age from 70 to 85. Individuals over 85, a rapidly increasing proportion of the population, are not included. In contrast, participants between the ages of 3–17 are studied in single year age cohorts. Given the substantial differences in cognition between people at age 18 and 69, extensive research will be needed to understand the utility of the toolbox's tests for longitudinal studies in aged samples.

A second large NIH-funded project involves assessment of executive cognitive function. Systematic definition of executive function has proved elusive. For instance, one review found that anywhere from one to four factors emerged as significant contributors to executive function (Royall et al., 2002), and these are variably sensitive to the aging process. In 2006, the NINDS funded the project “Domain Specific Tasks of Executive Function,” commonly known as EXAMINER, to develop a neuropsychological test battery which could reliably and validly assess domains of executive function (examiner.ucsf.edu). Preliminary evidence from 212 healthy adults over age 55 shows a significant negative correlation between a composite executive function score and age (Kramer, 2010). However, although executive function changes may be important contributors to cognitive aging, the EXAMINER project has not focused on other domains, especially those that most closely overlap with experimental paradigms useful in non-human species.

Substantial cognitive performance data has been collected by the NIA's Alzheimer's Disease Centers program since its inception in 1984. Since 2005, the Centers have collected a uniform data set of typical neuropsychological performance measures. This battery now also serves as the core cognitive assessment for another NIH collaborative effort, the Alzheimer's Disease Neuroimaging Initiative. The testing was intended to take no more than 30 min to administer, and includes assessments of (1) attention, (2) processing speed, (3) executive function, (4) episodic memory, and (5) language. Data on over 3600 cognitively normal subjects has been reported (Weintraub et al., 2009). Design of the battery was driven by pragmatic and disease-oriented considerations rather than by changes expected in healthy cognitive aging. Data is made freely available to investigators who request it (www.alz.washington.edu/WEB/researcher-home.html). However, there was no overt attempt to link the assessments to testing available in non-human species.

From the perspective of the medical provider, none of these battery approaches offers an ideal solution to office-based assessment of cognition. They are time-consuming and require special training or equipment. Additionally, copyright issues for some assessments, including the commonly used Mini-Mental State Examination, might become prohibitive in light of low reimbursement for these services by healthcare insurers. However, as the population of oldest adults grows dramatically over the coming decades, there will be increasing need for well-validated tools for assessment of cognition in aging and detection of diseases causing cognitive impairment. Public health agencies have recognized this need as well, and beginning in 2011, the US Centers for Medicare and Medicaid Services have made provision for reimbursement for assessment of cognitive function as part of an annual wellness visit.

The time therefore seems ripe for a coordinated and systematic approach to developing a library of tests useful to the researcher specifically interested in human cognitive aging, which can be directly linked to studies in non-human species and tap similar processes across multiple species. As noted above, domains of (1) associative memory, (2) recognition memory, (3) spatial and contextual memory, (4) working memory and executive function, and (5) visual perception offer the most promise. There is a rich body of human literature in each of these domains, and the influence of aging on performance is well supported. Specific experimental techniques for understanding the effects of aging in these and other domains will be reviewed by Alexander et al. (2012). We anticipate that a clearer understanding of the basis of cognitive aging in humans and non-human species will emerge from this approach. Additionally, gaps in knowledge that result from differences in experimental approaches can be identified, and used as a blueprint for development of new research assessments in either humans or non-human species. Improved understanding of the common physiologic basis and phenotypic expression of cognitive aging across species can also serve as the focal point for rationally designed and clinically practical tests, which would improve the ability of practicing physicians to differentiate the expected changes of healthy cognitive aging from more ominous patterns predictive of neurological disease.

## Challenges in human studies

Harmonizing the large bodies of work in humans and non-human species brings forward several challenges. Most obvious is the fact that human testing is almost always managed through the interface of language. While it may seem simplest to develop experimental tests that do not depend on verbal information, the validity of those tests for humans who developed and operate in a verbal world will require close examination. For instance, even among presumably non-verbal neuropsychological tests, factors such as culture and educational achievement significantly influence performance (Rosselli and Ardila, 2003).

Human subjects generally represent a much less homogeneous population than non-human experimental animals. Diseases, both age-related and not, and the effects of medications used to treat them, are much more difficult to control in human samples. Similarly, non-disease, non-cognitive changes with aging, such as declines in vision and hearing, have major impacts on cognitive performance. However, these may not be recognized by participants or researchers. The NIH Toolbox project has recognized the importance of these factors and is developing assessments of sensory, motor, and emotional function that will likely be useful in studies of human cognitive aging. In their companion paper in this issue Geldmacher et al. (2012) address the complex issues of identifying and excluding individuals with clinically relevant disease states from studies of humans, as well as other hard-to-control variables like gender, culture, and education.

Human studies are also complicated by different approaches to describing norms. There are few longitudinal studies of domain-specific cognitive performance in healthy elders. Cross-sectional data, either treating age as a continuous variable or grouping subjects into cohorts (e.g., age 60–69, 70–79, etc.) is more common. A particular problem with cross-sectional methods is the inability to determine whether a subject is manifesting subclinical findings of an incipient neurodegenerative disease.

Although a long-term goal of the McKnight Institutes' initiative is to assist clinicians to identify and manage cognitive changes in older adults, there are substantial differences in research and clinical applications of cognitive testing. Most researchers focus on a single domain of cognition, for instance breaking down a complex construct like memory into multiple component functions. In contrast, clinicians most often face complaints that span multiple domains. Even current brief cognitive assessment instruments are used in different ways in clinical and research paradigms. For instance, the Mini Mental State Exam may be used to establish an eligibility threshold for more detailed research assessment, whereas the clinician uses it as the whole of the cognitive assessment. Clinicians also have a need for tests that provide a high degree of discrimination between what is normal (or acceptable) and what represents disease, while researchers' needs may be met by tests that change predictably over time.

Two other emerging factors may change the role for age-sensitive cognitive tests in clinical settings over the next decade. One is the continued evolution of user-friendly, inexpensive, internet-based cognitive testing. There are currently many of these programs available for use in clinical research, and others aimed at self-improvement and cognitive fitness. Taken in conjunction with ever-increasing rates of computer literacy among those classified as elderly, additional new paradigms for cognitive assessment in aging can be anticipated. This is an opportunity as well as a challenge, since new computer-based cognitive tests can be modeled after existing assessments for non-human species and individualized to ameliorate age-related sensory changes (e.g., visual contrast sensitivity losses). Another important development is the release and undoubted further refinement of pathophysiologic biomarkers for age-related illnesses like Alzheimer's disease. A diagnostic test that a physician can order without the need for bedside cognitive assessment and without accounting for factors like age, gender, education, culture, and concomitant conditions will likely find a high rate of adoption.

In the companion articles in this special issue we will address critical aspects of age-related cognition in non-human species, and how they relate to the assessment of human cognitive aging. We will also address important considerations for interpreting studies of cognition in human subjects. We anticipate laying the groundwork for the development of a library of tests that can be applied across species, and suggest domains where new testing paradigms would advance the field. Finally, we hope to identify practical outcomes from this work that will allow clinicians to be more effective in characterizing cognitive changes in older adult patients presenting with memory concerns.

### Conflict of interest statement

The authors declare that the research was conducted in the absence of any commercial or financial relationships that could be construed as a potential conflict of interest.
